# CD30 expression and survival in extranodal NK/T-cell lymphoma: a systematic review and meta-analysis

**DOI:** 10.18632/oncotarget.24044

**Published:** 2018-01-08

**Authors:** Zihang Chen, Pujun Guan, Tong Shan, Yunxia Ye, Limin Gao, Zhi Wang, Sha Zhao, Wenyan Zhang, Li Zhang, Ling Pan, Weiping Liu

**Affiliations:** ^1^ Department of Pathology, West China Hospital, Sichuan University, Chengdu, Sichuan 610041, China; ^2^ Huaxi Magnetic Resonance Research Center, Department of Radiology, West China Hospital, Sichuan University, Chengdu, Sichuan 610041, China; ^3^ Department of Hematology, West China Hospital, Sichuan University, Chengdu, Sichuan 610041, China; ^4^ Institute of Public Health, Feinberg School of Medicine, Northwestern University, Chicago, Illinois 60611, USA

**Keywords:** CD30, extranodal natural killer/T-cell lymphoma, prognostic value, meta-analysis

## Abstract

**Background:**

The paradoxical reports about the prognostic value of the CD30 expression in extranodal NK/T-cell lymphoma (ENKTL) have restricted its further applications in clinical practice. To identify the common effects and the variation, we conducted this systematic review and meta-analysis.

**Methods:**

PubMed, MEDLINE, Embase, and Web of Science were searched between January 1975 and 31 January 2017. The pooled hazard ratio was used to estimate the effect of the CD30 expression on overall survival. Bias was assessed by prespecified criteria referring to Reporting Recommendations for Tumor Marker Prognostic Studies and Newcastle-Ottawa Scale.

**Results:**

Ten retrospective cohort studies with 310 patients are included. CD30 is associated with better overall survival significantly (HR 0.71, 95% CI 0.51 to 0.99, *I*^2^ = 0%). A greater effect is observed among studies including participants predominant in regional involvement (HR 0.31, 95%CI 0.13 to 0.76, *I*^2^ = 0%) compared with those in systemic involvement.

**Conclusions:**

This study indicates that the CD30 expression is significantly associated with better prognosis in ENKTL, especially for patients with regional lymphoma involvement.

## INTRODUCTION

Extranodal natural killer/T-cell lymphoma (ENKTL) is an aggressive, life-threatening lymphoproliferative disorder which is not only endemic in East Asia, Central and South America but also sporadic in Western countries, however, we still know little about the underlying biological nature involved in the management of it [1]. Several tools have already been used to predict the prognosis, such as International Prognostic Index (IPI), Korean Prognostic Index (KPI), Prognostic Index of Natural Killer Lymphoma (PINK) and prognostic nomogram. All of these tools are based on clinical features and laboratory data which could describe the overall impact on a human organism, but no pathological marker is taken into consideration [2–4]. Clinical features, while often useful for estimating the outcome, are too late for choosing positive and effective therapy. The study of the molecular change might remedy this defect.

Former research has demonstrated that CD30 expresses from non-neoplastic lymphoid tissue (some activated T and B immunoblasts) to classical Hodgkin lymphoma (cHL) and anaplastic large cell lymphoma (ALCL) [5, 9]; it has also been reported that CD30 expresses in 20% to 50%, even 70% patients with ENKTL [6]. Recently, brentuximab vedotin, one kind of antibody for CD30, has been applied to treat refractory cHL and ALCL and achieved dramatic clinical outcome [10]. Whether the anti-CD30 therapy could be applied to ENKTL is essential to the next foreseeable therapeutic promotion. Thus, it is important to understand the natural prognostic value of the CD30 expression in ENKTL.

Some studies implied that CD30 could be a candidate for estimating the survival of ENKTL patients, and might help choose suitable chemotherapy protocol or biotherapy [6–8]. However, the published research reported paradoxical results about the prognostic value of the CD30 expression in ENKTL. Thus, we aim to resolve the uncertainty situation by conducting a systematic review and meta-analysis of all published studies. In the discussion part, we especially focus on explaining our results on the basis of former molecular studies and potential treatment value of CD30 in ENKTL.

## RESULTS

### Study characteristics

**Figure 1:**
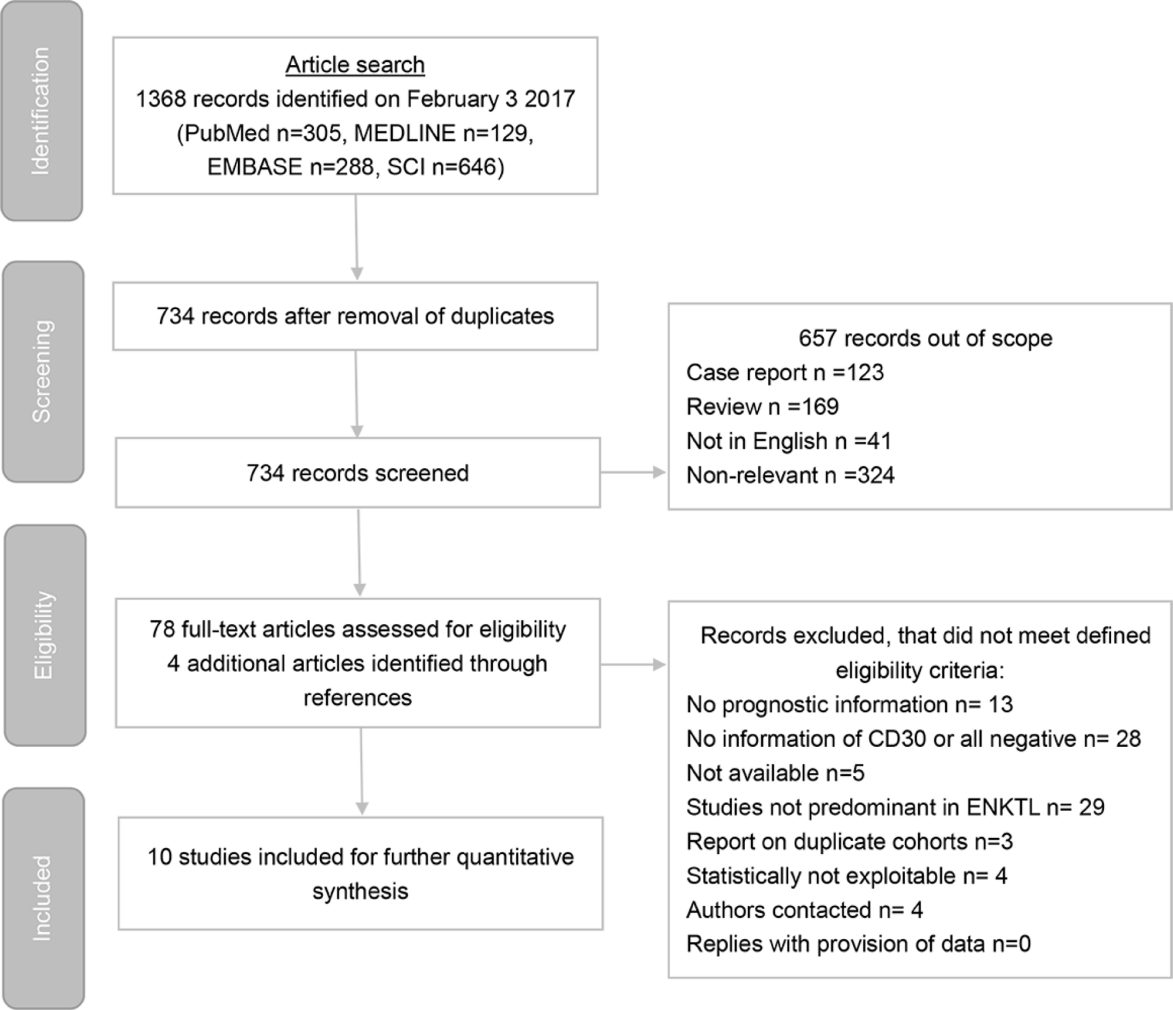
Preferred reporting items for systematic reviews and meta-analyses (PRISMA) flow chart of study identification process

Ten of 1368 searched studies meet our eligibility criteria (Figure 1) and the characteristics of studies are summarized in Table 1 (Supplementary Table 1 in details) [11]. Nine studies embed pure ENKTL cohorts; 1 study includes primary cutaneous ENKTL only and 3 include initial gastrointestinal involvement. Only one study includes mixed cohort (peripheral T cell lymphoma and ENKTL), and ENKTL is the majority type.

**Table 1:** Baseline characteristics of included studies

The first author	Year	Countries and Regions	*N*	N in survival analysis	Number of patients with extranasal involvement	Number of patients in stage I	Number of Patients with CD30 positive	Number of Patients with EBER positive	CT	RT	Surgery	Other therapy	No treating	Analysis method	HR estimation method
Mraz-Gernhard S [50]	2001	USA	30	27	27/27 (100%)	17/27 (63.0%)	7/27 (25.9%)	27/27 (100%)	20/27 (74.1%)	7/27 (25.9%)	3/27 (11.1%)	2/27 (7.4%)	4/27 (14.8%)	IHC	Calculated from raw data
Kuo TT [51]	2004	Taiwan	22	19	5/19 (26.3%)	19/19 (100%)	9/19 (47.3%)	19/19 (100%)	18/19 (94.7%)	16/19 (84.2%)	0/19 (0)	0/19 (0)	0/19 (0)	IHC	Calculated from raw data
Takashashi E [52]	2008	Japan	6	5	5/5 (100%)	1/5 (20.0%)	2/5 (40.0%)	5/5 (100%)	4/5 (80.0%)	2/5 (40.0%)	0/5 (0)	2/5 (40.0%)	1/5 (20.0%)	IHC	Calculated from raw data
Pongpruttipan T [53]	2012	Thailand	22	20	5/20 (25%)	13/20 (65.0%)	15/20 (75.0%)	20/20 (100%)	20/20 (100%)	3/20 (15.0%)	0/20 (0)	0/20 (0)	0/20 (0)	IHC	Calculated from raw data
Hong J [7]	2012	Korea	22	22	1/22 (4.5%)	7/22 (31.8%)	8/22 (36.4%)	22/22 (100%)	21/22 (95.5%)	17/22 (77.3%)	0/22 (0)	0/22 (0)	0/22 (0)	IHC	Extrapolated
Kim WY [6]	2015	South Korea	72	71	24/71 (33.8%)	27/71 (38.0%)	26/71 (36.6%)	71/71 (100%)	64/71 (90.1%)	12/71 (16.9%)	3/71 (4.2%)	0/71 (0)	2/71 (2.8%)	IHC	Extrapolated
Fang JC [54]	2015	China	10	10	10/10 (100%)	1/10 (10.0%)	3/10 (30.0%)	10/10 (100%)	8/10 (80.0%)	0/10 (0)	10/10 (100%)	0/10 (0)	0/10 (0)	IHC	Calculated from raw data
Yu BH [55]	2015	China	55	18	18/18 (100%)	8/18 (44.4%)	8/18 (44.4%)	18/18 (100%)	13/18 (72.2%)	0/18 (0)	13/18 (72.2%)	0/18 (0)	5/18 (27.8%)	IHC	Calculated from raw data
Kim SH [56]	2016	South Korea	59	46	NR	6/46 (13.0%)	NR	NR	30/30 (100%)	9/30 (30.0%)	0/46 (0)	0/30 (0)	0/46 (0)	IHC	Reported in text
Hu LM [57]	2017	Japan	12	7	7/7 (100%)	2/7 (28.6%)	3/7 (42.9%)	7/7 (100%)	6/7 (85.7%)	0/7 (0)	7/7 (100%)	1/7 (14.3%)	0/7 (0)	IHC	Calculated from raw data

CT: chemotherapy, RT: radiotherapy, NR: not report, EBER: EBV encoded RNA, HR: hazard ratio, IHC: immunohistochemistry.

In general, the 10 studies include 310 patients. 245 patients are reported both survival and CD30 data. There are 84 females and 161 males (male: female = 1.92); 1 study only includes female patients. The age is ranged from 8 to 97 years old. All of the studies evaluate CD30 by IHC; the dilution is ranged from 40 to 100, while 5 investigations do not report it. The CD30 positive rate is 40.7% (81/199) and EBER positive rate is 100% (199/199). Extranasal involvement is presented in 84 patients (84/199, 42.2%) and 101 patients are assessed as regional involvement (stage I) (101/245, 41.2%); 3 studies include more than 50% patients of ENKTL in stage I while other 3 studies provide less than 20%. Seven of the 10 investigations provide the number of patients with B symptoms and elevated LDH; 3 studies include more than 50% of patients with B symptoms and 4 provide more than 50% of patients with elevated LDH. Majority patients receive chemotherapy (204/229, 89.1%), while parts of patients accept radiotherapy (66/229, 28.8%). The operation is performed on 31 patients (31/229, 13.5%) who are mainly from 5 studies which focus on primary intestinal or cutaneous ENKTL. A few patients receive other therapies such as the stem cell mobilization, the autologous hematopoietic stem cell transplantation and etc. (5/229, 2.2%). Twelve patients are not treated (12/229, 5.2%).

Two studies provide HRs; 6 studies supply individual data to calculate HRs and 95% CIs. In other 2 studies, HRs are extrapolated from Kalan-Meier curves. One of them include 55 patients with individual data but 37 patients were excluded from the survival analysis since unmeasured CD30 or loss to follow-up.

Four studies are evaluated as low-risk of bias; 6 studies are assessed as high-risk of bias (Table 2, Supplementary Table 2 in details).

**Table 2:** Risk of bias assessment

The first author	Selection bias	Performance bias	Measurement bias	Attrition bias	Reporting bias	Other potential sources of bias	Risk of bias
Mraz-Gernhard S [50]	High	High	Low	Low	Low	Low	High
Kuo TT [51]	Low	Low	Low	Low	Low	Low	Low
Takashashi E [52]	High	Low	Low	Low	Low	Low	High
Pongpruttipan T [53]	Low	Low	Low	Low	Low	Low	Low
Hong J [7]	Low	Low	High	Low	Low	Low	High
Kim WY [6]	Low	Low	Low	Low	Low	Low	Low
Fang JC [54]	Low	High	Low	Low	Low	Low	High
Yu BH [55]	Low	Low	Low	Low	Low	Low	Low
Kim SH [56]	Low	Low	Low	High	Low	Unclear	High
Hu LM [57]	High	High	Low	Low	Low	Low	High

### Overall analysis and sensitivity analysis

**Figure 2:**
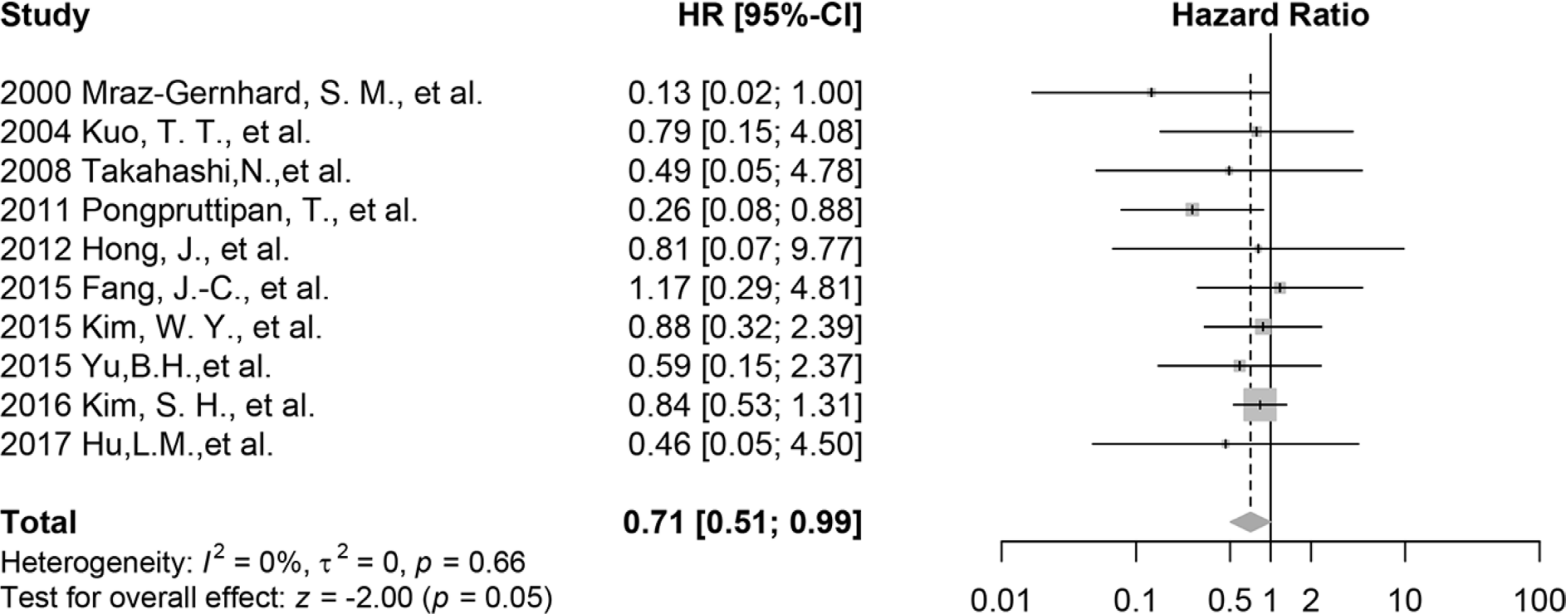
Forest plot of the effect of CD30 expression on survival, all 10 included studies

The meta-analysis of all included research indicates that CD30 is related to the better overall survival significantly (HR 0.71 95% CI 0.51 to 0.99, *p* = 0.05, *n* = 245; Figure 2) with low heterogeneity (*I*^2^ = 0%, *t*^2^ = 0, *p* = 0.66). Cross-Validation demonstrates that only removing the mixed cohort reported by Kim, S. H. *et al*. provides a smaller HR of 0.58 (95% CI 0.35 to 0.96, *p* = 0.04, *n* = 199, *I*^2^ = 0; Supplementary Figure 1), which reflects the effects from all pure ENKTL cohorts. When omitting other studies, mean HRs are from 0.69 to 0.77; the corresponding 95% CIs are depicted in Supplementary Figure 2.

### Subgroup analysis

**Figure 3:**
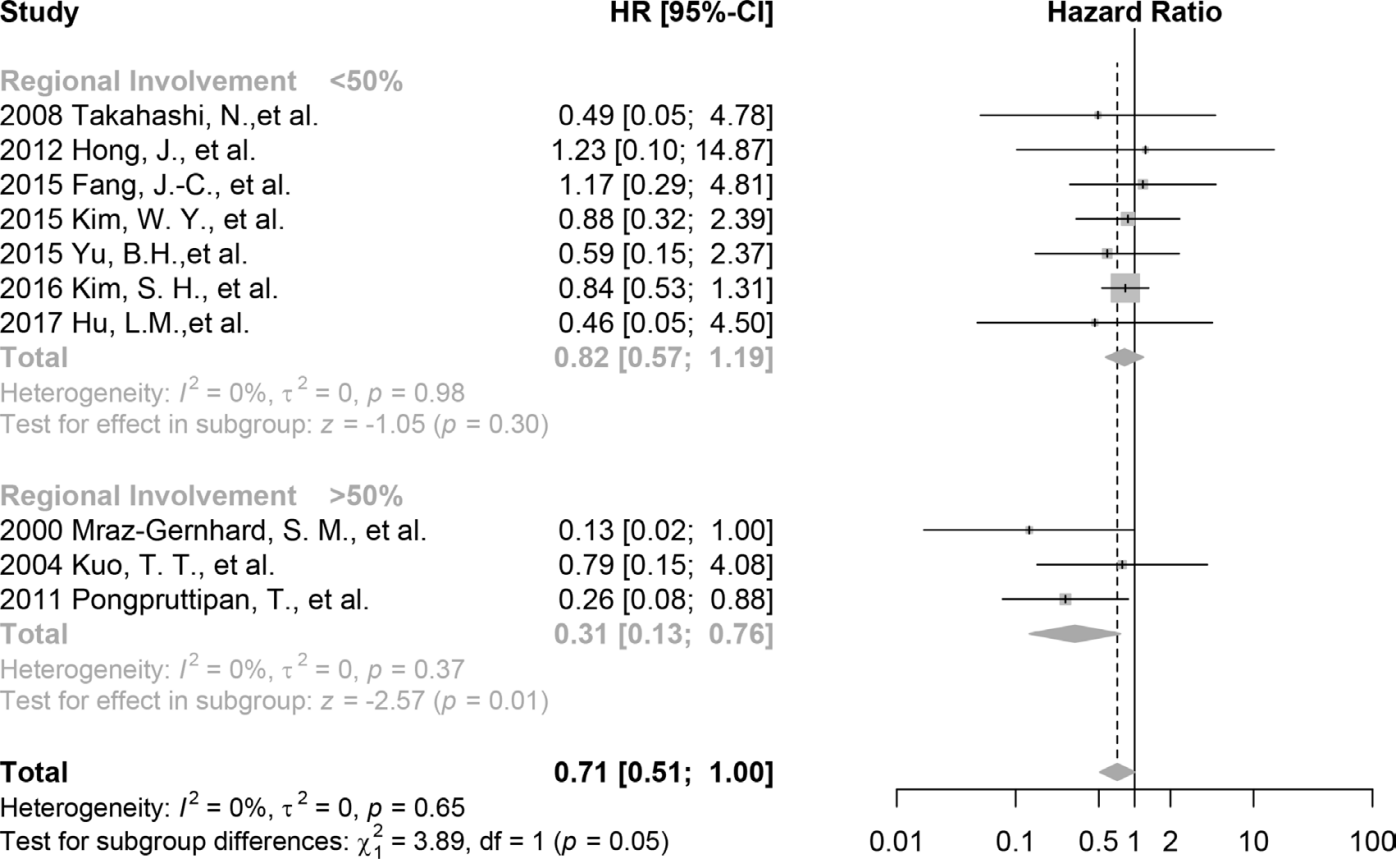
Forest plot of the effect of CD30 expression on survival stratified by tumor involvement, including studies with >50% patients with regional involvement and studies with <50% patients with regional involvement

The effect of the CD30 expression on overall survival is larger among studies included participants predominant in regional involvement (HR 0.31, 95%CI 0.13 to 0.76, *p* = 0.01, 3 studies, 66 patients, *I*^2^ = 0%) compared with those in systemic involvement (HR 0.82, 95% CI 0.57 to 1.19, *p* = 0.30, 7 studies, 179 patients, *I*^2^ = 0%) (Figure 3). The difference is statistically significant (chi^2^ = 3.89, *p* = 0.05). This result is consistent with the overall and the pure ENKTL analyses with a greater effect.

The effect of the CD30 expression on patient survival is larger in investigations that included patients predominant in nasal involvement (HR 0.45, 95%CI 0.18 to 1.12, *p* = 0.09, 3 studies, 61 patients, *I*^2^ = 0%) compared with those of primary gastrointestinal involvement (HR 0.75, 95%CI 0.30 to 1.87, *p* = 0.54, 3 studies, 35 patients, I^2^ = 0%) and other site involvement (HR 0.77, 95%CI 0.52 to 1.15, *p* = 0.20, 4 studies, 149 patients, *I*^2^ = 8%) (Supplementary Figure 3). However, the difference is not statistically significant (*p* = 0.09). Thus it might demonstrate a trend toward the benefit from the CD30 expression.

The prognostic value of CD30 is not significant in the subgroup, B symptoms (test for subgroup differences: chi^2^ = 0.06, *p* = 0.81, Supplementary Figure 4) and LDH (test for subgroup differences: chi^2^ = 0.02, *p* = 0.88, Supplementary Figure 5).

The prognostic value of CD30 tends to be larger in studies that assessed as low risk of bias (HR 0.58, 95%CI 0.31 to 1.08, *p* = 0.09, 4 studies, 128 patients, *I*^2^ = 0%) than those of high risk of bias (HR 0.78, 95%CI 0.52 to 1.16, *p* = 0.22, 6 studies, 117 patients, I^2^ = 0%) (Supplementary Figure 6). The difference between the groups is not statistically significant (*p* = 0.43).

### Small-study effects

The possible small-study effects (publication bias) are qualitatively visualized using funnel plot and quantified by Egger’s regression. There is no evidence for significant small-study bias (*t* = −1.31, *p* = 0.23; Supplementary Figure 7).

## DISCUSSION

This is the first systematic review and meta-analysis aiming to clarify the prognostic value of the CD30 expression in ENKTL, which includes 10 studies and more than 240 participants. This study indicates that the CD30 expression is a favorable prognostic marker in ENKTL. This effect is measured as a relative decrease in hazard of death of 41%, with up to a 223% decrease when studies of regional involvement are included. These results are also consistent with pure ENKTL cohort. Thus, CD30 is a biomarker candidate for predicting patients’ prognosis.

Earlier conflicting results might have several possible reasons. As shown in subgroup analysis, the first reason might be that different studies include patients predominant in different stages. The more early-stage patients a study includes, the greater CD30 benefit could be observed. The effect of CD30 might be masked by increasingly abnormal molecular events and gene instability when tumor goes to the late stage. The second reason might be different therapeutic tactics. According to Kim *et al.* applying anthracycline-based chemotherapy might also weaken the CD30 impacts [6]. Other chemotherapeutic drugs like L-asparaginase could also reduce the difference of survival time between CD30 positive and negative patients [12]. Whether surgery or radiotherapy have impact need to be studied further. We don’t group these studies by therapeutic methods because of insufficient data; it is a limitation of the meta-analysis. The third reason might be some studies have small sample sizes. Many point-estimation of HR was smaller than 1, but the 95% CIs covered 1. It could be improved when more patients are involved in one study and also partly improved thorough pooled estimation of HRs from many studies as this study did. So the stage and treatment method need to be fully reported in future studies with large sample size.

The evidence from previous molecular studies could also help us understand the results of this meta-analysis. The CD30 expression might be an early molecular event [13, 14]. Epstein Barr Virus (EBV), which is believed as a key component in the etiology of ENKTL, induce the CD30 expression by infection and transformation of lymphocytes, because EBV integration site (EBVS1) has been demonstrated close to the CD30 locus (at 1p35) of human which may activate the CD30 expression [15]. In addition, EBV-latent gene products such as latent membrane protein-1 (LMP-1) and EBNA2 upregulate CD30 on the surface of infected cells [16]. Inflammation from EBV infection which has also been observed as a cause of the CD30 expression in bystander neoplastic cells and elevated soluble CD30 [14]. This description has been also supported by other studies investigating inflammatory disorders such as systemic lupus erythematosus and inflammatory bowel disease [17, 18]. That might be why the effect of the CD30 expression is more obvious among studies included participants predominant in the early stage.

**Figure 4:**
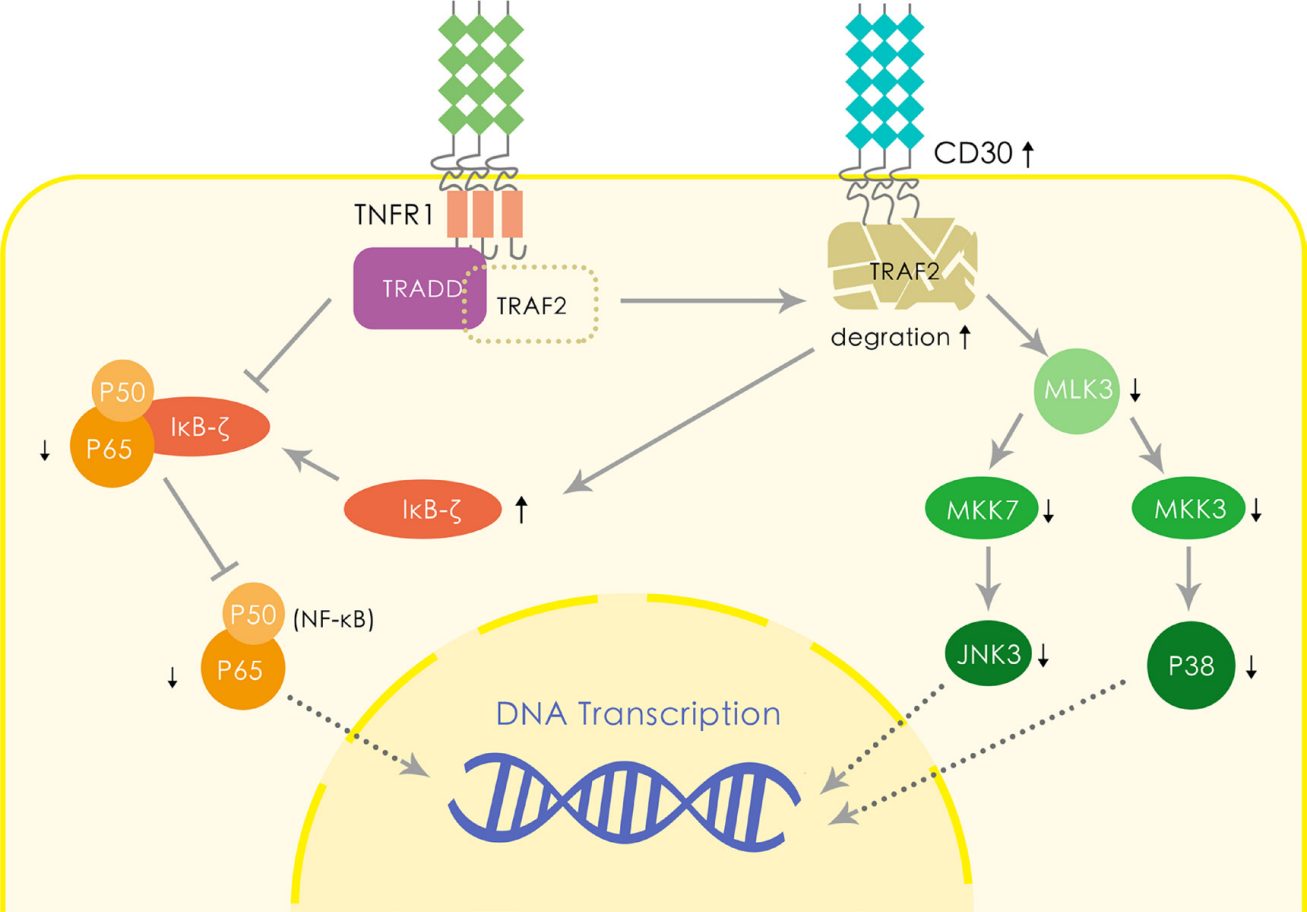
Hypothetical molecular mechanism of CD30 leading to the favorable prognosis of ENKTL

Two probable hypotheses based on published molecular biology results could explain the favorable impact of CD30 on the prognosis of ENKTL (Figure 4). Firstly, the CD30 expression increases TRAF2 degradation thus downregulates NF-κB activation, which has been observed related to the poor survival in patients with ENKTL [19–21]. The expression of CD30 exposes NF-κB to TRDD and downregulates the NF-κB pathway [22, 23]. In addition, CD30 enhances IκB-ζ expression, resulting in negative impact on NF-κB activation [24]. Therefore, we conjecture such mechanism may also play a part in ENKTL and lead to a favorable survival via downregulating NF-κB. Secondly, CD30 downregulates MLK3/MKK7/JNK3 signaling pathway and MLK3/MKK3/p38 signaling pathway [25]. MLK3 has been demonstrated to be a potent factor associated with the aggressive course in many malignancies; CD30 induced TRAF2 degradation reduces MLK3 expression, resulting in downregulation of JNK3 expression via decreased MKK7 expression [25, 26]. On the other hand, the CD30 expression reduces p38 synthesis via suppressing MKK3 [27]. Previous investigations reported that suppression of MLK3/MKK7/JNK3 pathway contributes to chemotherapy-induced apoptosis in many tumors [28–30]. Decreased expression of p38 also increases cell sensitivity to apoptosis induced by caspase-8 [31]. Other possible explanations include that CD30 might be related to the STAT3 mutation which prevents JAK3 pathway from activation and results in a better prognosis [32–36], which has been shown to have tight relation with proliferation in normal human cells. Further studies are needed to verify and elucidate it.

This study is timely because the CD30 expression probably helps improve survival by guiding treatment [37]. A novel anti-CD30 antibody, brentuximab vedotin, was approved by Food and Drug Administration to treat refractory cHL and ALCL in 2011, which throws the light on therapeutic tactics for other CD30 positive lymphomas. Two successful cases in treating refectory ENKTL with brentuximab vedotin have been reported with relatively low CD30-positive rate (nearly 30%) compared with cHL and ALCL (usually more than 90%) [12, 38]. However, the cut-off of the CD30-positive rate for treating ENKTL in using brentuximab vedotin has been ambiguous and need to be investigated. Lots of aspects might contribute to the anti-CD30 therapy, such as the mechanism anti-CD30 of ENKTL and bystander killing phenomenon (neighborhood cells which are negative in CD30 surrounding the CD30 positive tumor cells also be killed since the CD30 positive neoplastic cells secret CD30-expressing extracellular vesicles binding to CD30 ligands on bystander cells) [39, 40]. In addition, for patients with low CD30-positive rate ENKTL, epigenetic therapy with drugs like vorinostat and cladribine could be applied to induce the CD30 expression in neoplastic cells before treating with anti-CD30 antibody in order to achieve a better outcome [41].

This study also gives us a hint that anti-CD30 clinical trials need carefully designing because there might be a chance to misestimate the effect of anti-CD30 therapy in ENKTL due to that 1) some ENKTL patients are CD30 negative, and 2) the positive rate is various. However, these effects could not be observed in trails of ALCL and cHL since most of them express CD30 in a rather homogeneous way compared with ENKTL [42, 43].

There were several limitations that may affect the validity of our findings. Firstly, despite conducting an extensive search strategy including potential clinical-pathological studies of ENKTL and performing the test for small-study effect, however, we could not totally exclude small-study effect as a possible explanation of our results since only 10 studies with partly small sample size were included. Secondly, the number of studies which are evaluated as high-risk of bias (*n* = 6) were relatively more than studies of low-risk of bias (*n* = 4) although the summed sample size of all low-risk studies (*n* = 128) is larger than that of high-risk studies (*n* = 117). In addition, the CD30 expression was only assessed as “present” or “absent” other than expression rate due to data limitations, which prevents us from investigating the prognostic value of CD30 deeply. Besides, the variety of dilution of antibody applying to IHC staining serves as another potential confounder since it was unreported in half of the included studies. Moreover, as mentioned above, treatment tactics (chemotherapy regimen, surgery, radiotherapy), especially for chemotherapy regimen and the dose of radiotherapy, may affect the outcome, which may lead to an underestimate of the actual effect of the CD30 expression. Finally, only one of our studies were investigated in the western country while the rest of studies were researched in Asia, which may suggest an ethical bias but was unavoidable since ENKTL is prevalent in Asia.

In summary, ENKTL remains a rare type of life-threatening non-Hodgkin lymphoma of which immunophenotype information for guiding patient management is still limited. This study indicates that the CD30 expression has a significantly positive prognostic impact on patients with ENKTL, which could also serve as a target candidate. High-quality studies with large sample size cohorts in appropriate subgroups based on the rate of the CD30 expression are needed to confirm our result and dig into the molecular mechanism deeply.

## METHODS

### Search strategies and selection criteria

The databases PubMed, MEDLINE, Embase, and Web of Science were searched for potentially suitable studies published between January 1975 and 31 January 2017 using mesh terms “Antigens, CD30” and “Lymphoma, Extranodal NK-T-Cell” as well as text words including “TNFRSF8” or “Ki-1” or “Ber H2” or “CD30” and “natural killer/t cell lymphoma” or “natural killer-t cell lymphoma” or “NK-T cell lymphoma” or “NK/T cell lymphoma”. We later used “clinicopathological” and aforementioned ENKTL terms to extend the search. Conference papers were not screened or excluded on purpose, however, if conference paper met the inclusion criterion, we tried to get the formal published version for further assessment.

Two reviewers (CZH and GPJ) scanned the entire paper list independently. After removing duplicates, titles and abstracts were used for whether to retrieve the full text and it would be used for further identifying eligible papers. References from related research and reviews were used to additionally screen for studies that might be missed by the strategy above.

The inclusion criteria: 1) the language of original articles were English. 2) NK/T cell lymphoma met diagnosis based on the WHO classification of tumors of hematopoietic and lymphoid tissues (3rd or 4th edition). Mixed cohort would be included if ENKTL is the majority type in the cohort. 3) The CD30 was measured by immunohistochemistry (IHC) or other proper methods. 4) The hazard ratio (HR) and the corresponding 95% confidence interval (CI) (or data sufficient to calculate them) were reported. If the articles provided inadequate data, we contacted the authors by emails for required information.

The exclusion criteria: 1) Case reports, reviews, original articles based on cell line or animal experiment data 2) Including all patients of CD30 positive or negative 3) Duplicate cohort (If the same research groups published more than one paper including overlapping cohort, we chose the latest paper to avoid duplication).

### Data extraction and endpoint

We reviewed all 10 eligible studies and extracted variables into a standardized data extraction form as follows: name of the author; publication year; country or area where study was conducted; number of included patient and patients in survival analysis; number of patients of CD30 positive; number of patients of EBV encoded RNA (EBER) positive; number of patients in each stage; treatment strategy (chemotherapy, radiotherapy, surgery, other therapy or received no treatment); CD30 assay method (all specimens in eligible studies were evaluated by IHC); antibody type of IHC; dilution for IHC; HR estimation method and HRs with 95% CI or data for calculating HRs where available. We also extracted the number of patients with extranasal involvement, the ratio of male to female, median age, number of patients suffered from B symptoms, number of patients with elevated LDH for each study in order for subgroup analysis. The endpoint was chosen as overall survival because no sufficient studies for evaluating progression-free survival or disease-free survival.

### Risk of bias, study quality assessment

All studies were evaluated for risk of bias for study estimate of the significance of CD30 of survival from six aspects (study subjects, biomarker measurement, outcome measurement and account, subject attrition, analysis approach) consulted for reporting recommendations for tumor marker prognostic studies (REMARK) and Cochrane principles for quality assessment for prognostic studies [44–46]. The Newcastle-Ottawa Scale was also used as a reference [47]. (All evaluated subclasses for risk can be found in supplementary table, Supplementary Table 2) Risk of bias for each aspect was marked as high, low or unclear according to appraisal criteria. The overall risk of bias for the study was evaluated as high if “high” ≥1.

### Statistical analysis

All the included studies were classified as studies that reported A) HR with 95% CI, B) original individual data with the CD30 expression and survival information and C) corresponding Kaplan-Meier curves. For group A, HRs were extracted when calculate by univariate analysis, because often different models were used in multivariable analysis and the corresponding coefficient could not be directly pooled together. In group B, we use package “survival” (version 2.40–1) in R statistical software to directly calculate HRs and their variance. And in group C, we use Parmar method and Tierney Excel program for estimating HRs from Kaplan-Meier curves [48, 49]. The log (HR) and SE were all calculated use Tierney Excel chart as well.

To estimate overall HR and CIs, the generic inverse variance method was used in R package “meta” (version 4.8–1). We chose a fix-effects model to get pooled HR and 95%CI. Heterogeneity was tested using chi^2^ test with alpha = 0.1 or quantitatively by I^2^ statistic, with 30% as the cut-off value for substantial heterogeneity.

The subgroup analysis was performed to assess the impact of potential bias on overall survival by repeating the pooling analysis in the following subgroups: 1) different risks of the study, 2) regional involvement versus systemic involvement, 3) frequent B symptoms suffering versus rare B symptoms, 4) elevated LDH versus normal ranged LDH. Moreover, an exploratory subgroup was divided as nasal ENKTL versus primary intestinal involvement ENKTL versus mixed involvement (nasal and extranasal involvement) ENKTL study populations. The impact of induvial studies on overall effects was checked by leave-one-out cross-validation to avoid the results were driven by any single study. Publication bias was qualitatively visualized using funnel plot and quantified by Egger’s regression in R statistical software, package “meta” (version 4.8–1). All *p* ≤ 0.05 was considered statistically significant except the test of heterogeneity.

## SUPPLEMENTARY MATERIALS FIGURES AND TABLES







## Abbreviations

ENKTL: extranodal natural killer/T-cell lymphoma
EBV-T/NK-LPD: Epstein-Barr virus-associated T/natural killer cell lymphoproliferative disorder
IPI: International Prognostic Index
KPI: Korean Prognostic Index
PINK: Prognostic Index of Natural Killer Lymphoma
CD30: Cluster of differentiation 30
TNFRSF8: tumor necrosis factor receptor superfamily member 8
cHL: classical Hodgkin lymphoma
ALCL: anaplastic large cell lymphoma
IHC: immunohistochemistry
CRT/X: Chemotherapy and/or radiotherapy
HR: hazard ratio
LDH: lactate dehydrogenase
REMARK: reporting recommendations for tumor marker prognostic studies
SE: standard error
CIs: confidence intervals
EBVS1: EBV integration site
LMP-1: latent membrane protein
EBNA2: Epstein-Barr Nuclear Antigen 2
TRAF2: TNF receptor-associated factor 2
NF-κB: nuclear factor kappa-light-chain-enhancer of activated B cells
TRADD: TNFR1-associated death domain protein
IκB: inhibitor of NF-κB
MLK3: Mixed lineage kinase 3
MKK7: mitogen-activated protein kinase kinase 7
MKK3: mitogen-activated protein kinase kinase 3
JNK3: c-Jun N-terminal kinase 3
STAT3: Signal transducer and activator of transcription 3
