# Supplementary material for: CD30 expression and survival in extranodal NK/T-cell lymphoma: a systematic review and meta-analysis
Source: Oncotarget. 2018 Jan 8;9(23):16547–56. doi: 10.18632/oncotarget.24044 (PMC5893261; doi:10.18632/oncotarget.24044)
Supplement: Supplementary file 1 [file oncotarget-09-16547-s001.pdf]

CD30 expression and survival in extranodal NK/T-cell lymphoma: a systematic review and meta-analysis

SUPPLEMENTARY MATERIALS

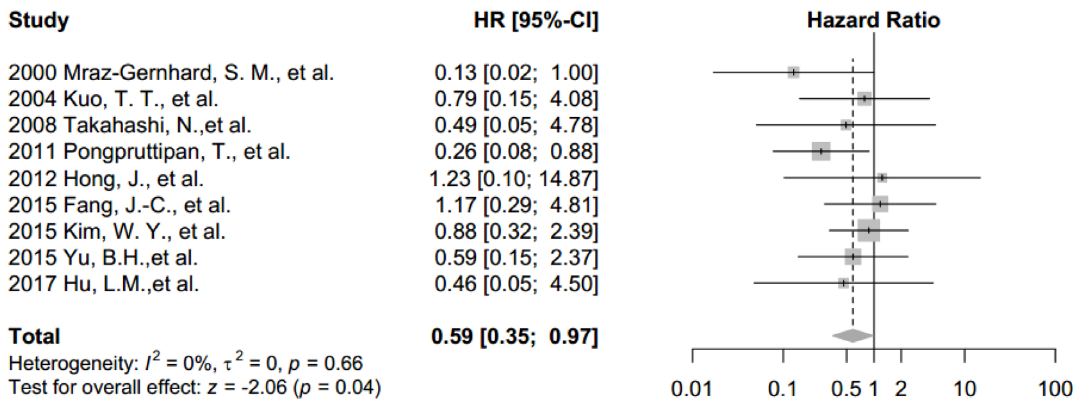

Supplementary Figure 1: Forest plot of the effect of CD30 expression on patient survival stratified by pure ENKTL cohorts.

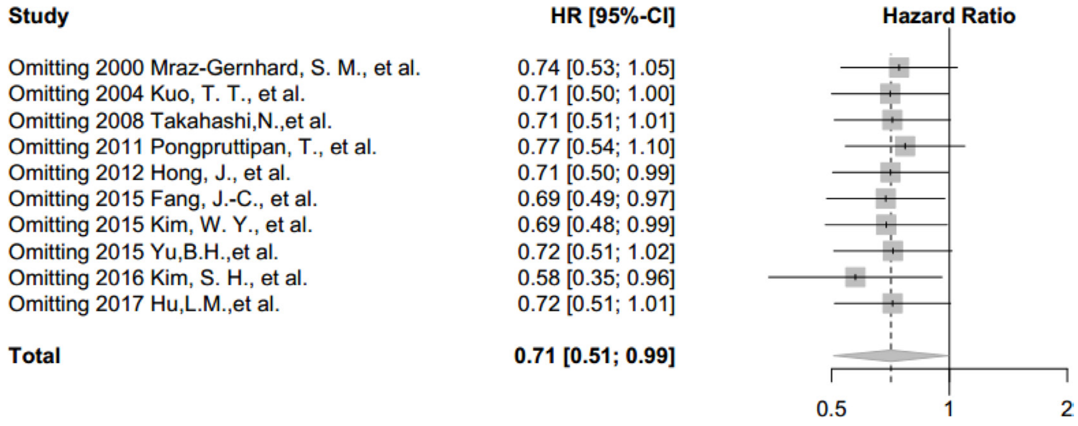

Supplementary Figure 2: Sensitivity analysis (leave-one-out) of the effect of CD30 expression on ENKTL.

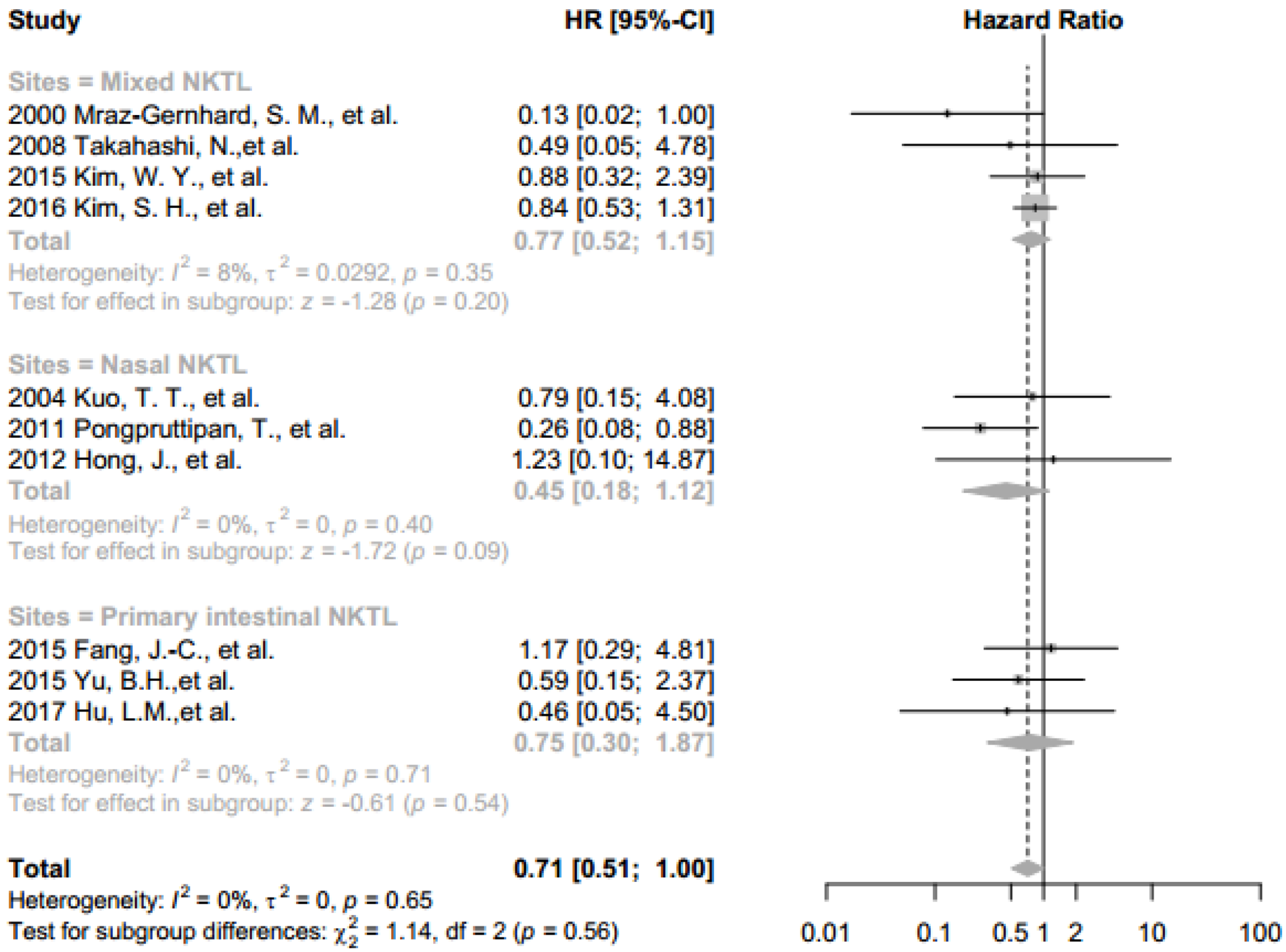

Supplementary Figure 3: Forest plot of the effect of CD30 expression on patient survival stratified by tumor site.

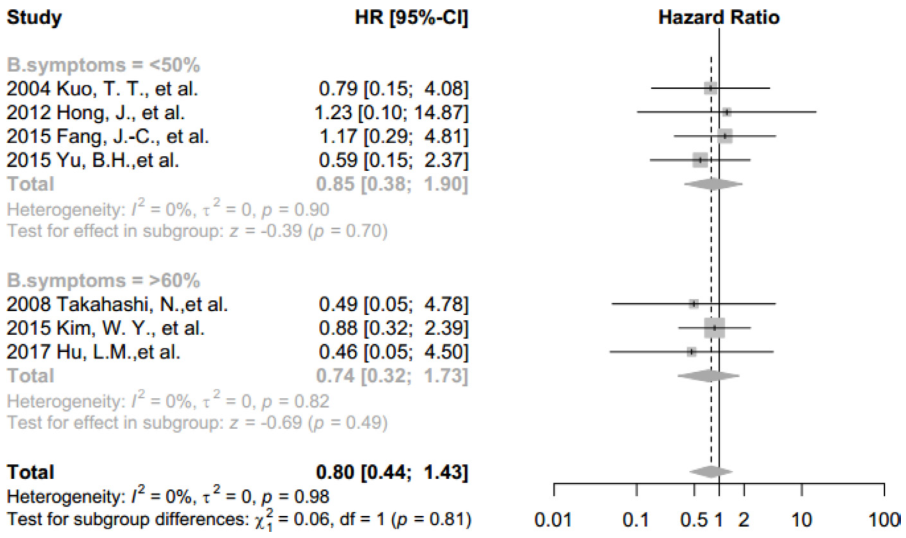

Supplementary Figure 4: Forest plot of the effect of CD30 expression on patient survival stratified by B symptoms.

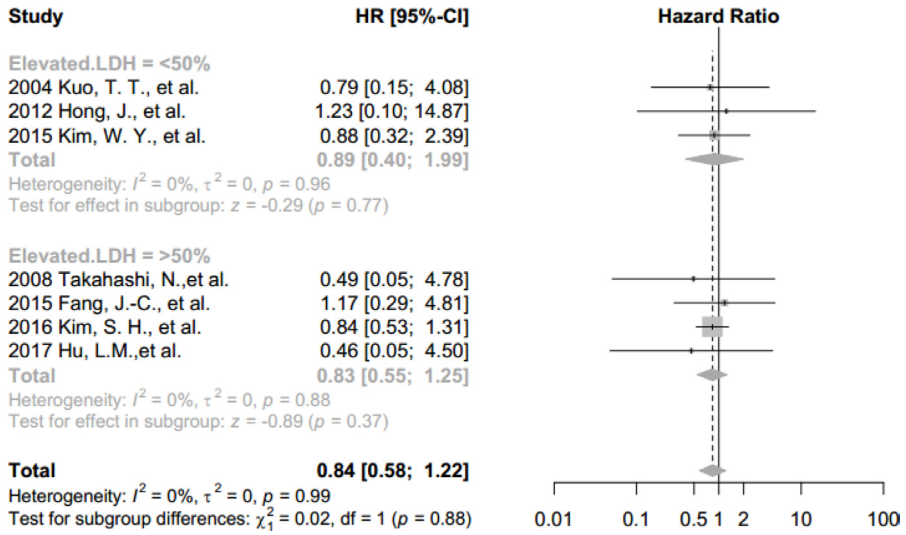

Supplementary Figure 5: Forest plot of the effect of CD30 expression on patient survival stratified by elevated LDH.

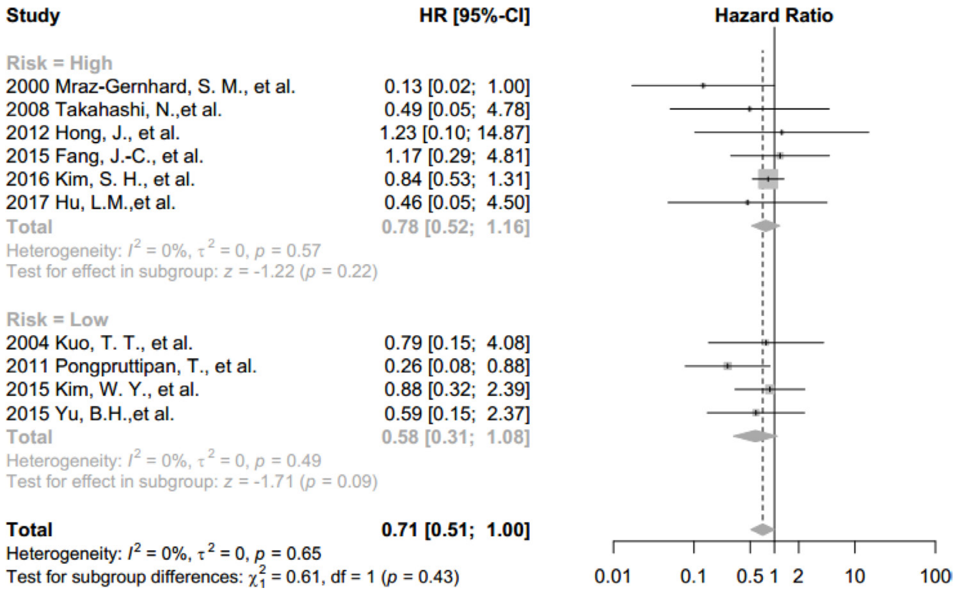

Supplementary Figure 6: Forest plot of the effect of CD30 expression on patient survival stratified by risk of bias.

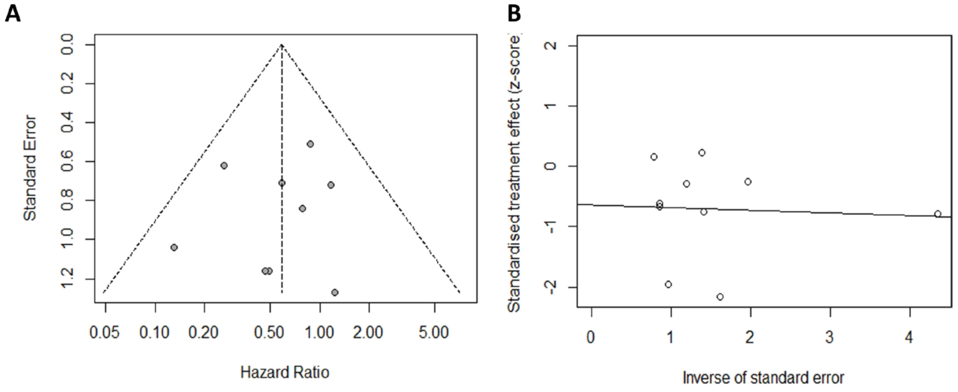

Supplementary Figure 7: Funnel plot for meta-analysis of 10 studies without small-study effect (**A**) and Radial plot including regression line (solid line) for Egger's test (**B**). Each point represents a separate study. There is no evidence for significant small-study bias.

Supplementary Table 1: Clinicopathological features of included studies. See Supplementary_Table_1

Supplementary Table 2: Risk of bias assessment in details. See Supplementary_Table_2
